# Molecular Epidemiology of Tuberculosis in British Columbia, Canada: A 10-Year Retrospective Study

**DOI:** 10.1093/cid/cix906

**Published:** 2017-10-21

**Authors:** Jennifer L Guthrie, Clare Kong, David Roth, Danielle Jorgensen, Mabel Rodrigues, Linda Hoang, Patrick Tang, Victoria Cook, James Johnston, Jennifer L Gardy

**Affiliations:** 1 School of Population and Public Health, University of British Columbia; 2 British Columbia Centre for Disease Control Public Health Laboratory; 3 British Columbia Centre for Disease Control; 4 Department of Pathology and Laboratory Medicine, University of British Columbia, Vancouver, Canada; 5 Department of Pathology, Sidra Medical and Research Center, Doha, Qatar; 6 Respiratory Medicine, University of British Columbia, Vancouver, Canada

**Keywords:** MIRU-VNTR genotyping, tuberculosis, molecular epidemiology, population structure

## Abstract

**Background:**

Understanding regional molecular epidemiology allows for the development of more efficient tuberculosis prevention strategies in low-incidence settings.

**Methods:**

We analyzed 24-locus mycobacterial interspersed repetitive-unit–variable-number tandem repeat (MIRU-VNTR) genotyping for 2290 *Mycobacterium tuberculosis* clinical isolates collected in the province of British Columbia (BC), Canada, in 2005–2014. Laboratory data for each isolate were linked to case-level clinical and demographic data. These data were used to describe the molecular epidemiology of tuberculosis across the province.

**Results:**

We detected >1500 distinct genotypes across the 4 major *M. tuberculosis* lineages, reflecting BC’s diverse population. Disease site and clustering rates varied across lineages, and MIRU-VNTR was used to group the 2290 isolates into 189 clusters (2–70 isolates per cluster), with an overall clustering rate of 42.4% and an estimated local transmission rate of 34.1%. Risk factors for clustering varied between Canadian-born and foreign-born individuals; the former had increased odds (odds ratio, 7.8; 95% confidence interval [CI], 6.2–9.6) of belonging to a genotypic cluster, although nearly one-quarter of clusters included both Canadian- and foreign-born persons. Large clusters (≥10 cases) occurred more frequently within the *M. tuberculosis* Euro-American lineage, and individual-level risk factors associated with belonging to a large cluster included being Canadian born (adjusted odds ratio, 3.3; 95% CI, 2.3–4.8), residing in a rural area (2.3; 1.2–4.5), and illicit drug use (2.0; 1.2–3.4).

**Conclusions:**

Although tuberculosis in BC largely arises through reactivation of latent tuberculosis in foreign-born persons, locally transmitted infections occur in discrete populations with distinct disease and risk factor profiles, representing groups for targeted interventions.

## Teaser

This 10-year retrospective study describes tuberculosis molecular epidemiology in a low-incidence setting. Approximately one-third of cases likely result from local transmission, largely – but not entirely – amongst the Canadian-born. Disease site and risk factors for clustering vary with lineage and birthplace.

As tuberculosis prevention and care programs in low-incidence, well-resourced settings look to accelerate progress toward elimination, it is clear that different interventions are required for different populations—whether enhanced screening and uptake of latent tuberculosis infection (LTBI) preventive therapy, or interventions aimed at accelerating diagnosis and reducing person-to-person transmission. To identify discrete groups of patients with tuberculosis and ultimately develop tailored interventions bespoke to each, we can leverage molecular genotyping methods such as 24-locus mycobacterial interspersed repetitive-unit–variable-number tandem repeat (MIRU-VNTR) analysis [1]. MIRU-VNTR is a polymerase chain reaction–based technique with high discriminatory power, often used to differentiate relapse from reinfection, detect laboratory cross-contamination events, and identify outbreaks and endemically circulating strains [2].

Canada has a low tuberculosis incidence rate of 4.4 cases per 100000 population, but among the provinces, British Columbia (BC) has one of the highest rates—6.3 cases per 100000 population [3]. More than 80% of BC’s patients with tuberculosis live in the Greater Vancouver region [3], home to approximately half of BC’s residents and the majority of BC’s foreign-born population [4]. This latter group represents 81% of BC residents with a diagnosis of tuberculosis [3], in whom active tuberculosis disease is generally thought to result from reactivation of LTBI acquired in the country of origin. Risk factors for tuberculosis disease in this group are probably markedly different from those in the patients whose disease results from a locally transmitted infection.

Previous population-based molecular epidemiological studies in Canada have focused largely on specific metropolitan areas [5–7] with few province-wide studies [8–10], and no provincial study has used 24-locus MIRU-VNTR; thus, we undertook a retrospective genotypic survey of all culture-positive tuberculosis diagnoses in BC from 2005 to 2014 to elucidate the patterns underlying tuberculosis transmission in BC.

## METHODS

### Study Setting and Design

The British Columbia Public Health Laboratory (BCPHL) of the British Columbia Centre for Disease Control (BCCDC) receives all *Mycobacterium tuberculosis* cultures for the province, and oversees routine diagnosis, and phenotypic drug sensitivity testing. Before 2014, genotyping was performed on request, with approximately 20% of isolates genotyped annually. We therefore designed a retrospective study to include all persons with culture-confirmed tuberculosis (79.5% of all 2915 diagnoses) residing in BC whose first *M. tuberculosis* isolate was received by the BCPHL from 2005 to 2014 (n = 2318). *Mycobacterium africanum* (n = 29)*, Mycobacterium bovis* (n = 3), and *M. bovis* bacilli Calmette-Guérin (n = 19) were excluded from the analysis; these are not commonly isolated at BCPHL and we do not expect local transmission. For individuals with a recurrence during the study period, we used data from their first episode only if isolates from their first and second episode had matching MIRU-VNTR (n = 11), and data from both episodes where MIRU-VNTR indicated reinfection (n = 2). Ethical approval was granted by the University of British Columbia (certificate H12-00910).

### Case Data

Individual-level clinical and demographic data were extracted from BCCDC’s Integrated Provincial Health Information System. We determined community type using the population density of the geographic service area in which each patient resided—metro (>190000), urban/rural (40001–190000), rural (10001–40000), or remote (≤10000). We used postal codes to obtain the corresponding census dissemination area for each patient and linked it to the 2006 Canadian marginalization index [11] to determine the deprivation index quintile, a neighborhood-level indicator of socioeconomic status. The deprivation index measures relative socioeconomic disadvantage of a dissemination area compared with the rest of Canada, reported as quintile values by dissemination area (quintile 1, least deprived; quintile 5, most deprived).

### Laboratory Analysis

All *M. tuberculosis* isolates were revived from BCPHL’s frozen archival stocks on Lowenstein-Jensen slants or in MGIT™ liquid medium (Becton-Dickinson). Phenotypic drug susceptibility results (isoniazid, rifampin, ethambutol, and streptomycin) were available for each isolate from routine testing on the BACTEC MGIT 460 or 960 (Becton-Dickinson). DNA was extracted using the MagMA Total Nucleic Acid Isolation Kit (Ambion).

Of the 2307 culture-positive isolates meeting study criteria (Supplementary Figure S1), 17 isolates had incomplete MIRU-VNTR or were unavailable for genotyping, leaving a total of 2290 isolates (99.3%), which were successfully genotyped using standard methods [1]. Major lineage was predicted for each isolate using TB-Insight’s CBN method [12]. Phylogenetic relationships within each lineage were visualized using a minimum-spanning tree (MST) in PHYLOViZ software (version 2.0) [13].

### Statistical Analysis

We defined a cluster as ≥2 patients with identical MIRU-VNTR patterns. We then estimated the odds ratio and 95% confidence interval (CI) for the distribution of patients by cluster status (clustered vs nonclustered) according to birthplace and other clinical and demographic variables. To examine factors associated with cluster growth we constructed a multivariable logistic regression model with cluster size—large (≥10 persons) versus small (<10 persons)—as the outcome, using backward elimination of factors identified in univariable analysis (*P <* .20) and Akaike’s information criterion minimization [14]. Because the variables (human immunodeficiency virus [HIV] status, illicit drug use and alcohol misuse) had >5% missing values, we performed Little’s test [15] to assess whether these data were missing completely at random. The results suggested no violation of this assumption, and missing values were unrelated to genotypic clustering (*P* > .05). To test the association between tuberculosis lineage and disease site, we used a χ^2^ test, and to examine time from immigration to active tuberculosis disease, as well as median age between clustered and nonclustered individuals, we used the Mann-Whitney *U* test. All analyses were executed with R software (version 3.3.1).

## RESULTS

Table 1 presents an overview of the demographic and clinical characteristics of culture-positive tuberculosis in BC. The median age was 52 years, with the highest proportion of diagnoses occurring in individuals aged 35–54 years. Male patients outnumbered female patients by a ratio of 1.4:1. Country of birth was available for 97.5% of patients, most of whom (73.7%) were foreign born. Although 78 countries were represented, most foreign-born patients with tuberculosis came from high-incidence settings [16], with 23.2% from India, 20.9% from Philippines, 18.5% from China, and 25.0% from other Asian countries. Most individuals (76.6%) lived in metro regions at the time of tuberculosis diagnosis.

**Table 1.** Demographic and Clinical Characteristics of Culture-Positive Patients With Tuberculosis in British Columbia, 2005–2014 (n = 2290)

Characteristic	Patients, No. (%)^a^
Age, y	
0–14	32 (1.4)
15–34	500 (21.8)
35–54	704 (30.7)
55–74	584 (25.5)
≥75	470 (20.5)
Male sex^b^	1329 (58.1)
Community type	
Metro	1753 (76.6)
Urban/rural	332 (14.5)
Rural	173 (7.6)
Remote	32 (1.4)
Birthplace^c^	
Canada	588 (26.3)
Foreign-born by continent^d^	
Asia	1437 (87.6)
Africa	79 (4.8)
Europe	69 (4.2)
Americas	45 (2.7)
Oceania	11 (0.7)
Time in Canada, y^e^	
<5	456 (28.6)
≥5	1141 (71.4)
Disease site	
Respiratory	1767 (77.2)
Nonrespiratory	363 (15.9)
Respiratory and ronrespiratory	160 (7.0)
Positive respiratory smear results^f^	1152 (62.1)
Cavitary disease present	315 (13.8)
Drug susceptibility	
MDR	18 (0.8)
INH-R (non-MDR)	173 (7.6)
HIV status	
Infected	103 (4.5)
Uninfected	1784 (77.9)
Unknown	403 (17.6)
Illicit drug use	
Yes	130 (5.7)
No	1639 (71.6)
Unknown	521 (22.8)
Alcohol misuse	
Yes	125 (5.5)
No	1656 (72.3)
Unknown	509 (22.2)
Material deprivation index^g^	
Quintile 1 (least deprivation)	273 (12.5)
Quintile 2	418 (19.2)
Quintile 3	529 (24.3)
Quintile 4	529 (24.3)
Quintile 5 (most deprivation)	427 (19.6)

Abbreviations: HIV, human immunodeficiency virus; INH-R, isoniazid resistant; MDR, multidrug- resistant tuberculosis (resistant to isoniazid and rifampin).

^a^Percentages have been rounded and may not total 100%.

^b^One transgender/gender-unknown patient excluded from analysis.

^c^Data unavailable in 57 patients.

^d^Data unavailable in 4 patients.

^e^Data unavailable in 48 patients.

^f^“Other respiratory” sites (eg, pleura) were excluded.

^g^Data unavailable in 114 patients.

With respect to clinical characteristics, most patients (77.2%) had respiratory tuberculosis, and of these, 16.3% of patients were characterized as having cavitary disease based on chest radiography. Of the patients for whom HIV status was known (82.4%), 103 were HIV infected. A small fraction of patients were recorded as using drugs (5.7%) or alcohol (5.5%). Phenotypic drug susceptibilities were available for all genotyped isolates, with multidrug-resistant (MDR) isolates defined as those with resistance to at least isoniazid and rifampin (18 isolates; 0.8%) (Supplementary Table S1).

### Lineage Analysis

**Figure 1.**
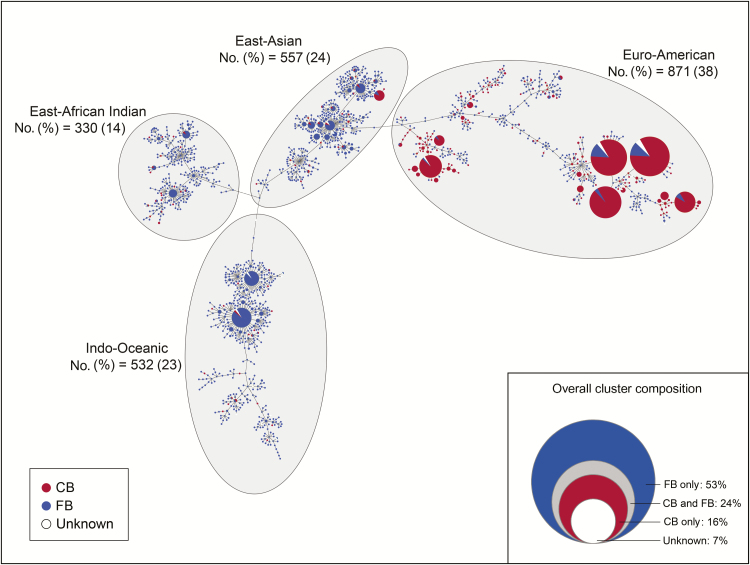
Minimum spanning tree analysis of 24-locus mycobacterial interspersed repetitive-unit–variable-number tandem repeat (MIRU-VNTR) genotyping for *Mycobacterium tuberculosis* isolates in British Columbia (2005–2014). The size of each circle is proportional to the number of isolates. Classification of strains by birthplace is visualized with color coding. The inset demonstrates overall cluster composition with respect to birthplace; relative frequency of clusters that were exclusively Canadian born (CB), exclusively foreign born (FB), Canadian born and foreign born (CB and FB), or included ≥1 isolate for which the patient’s birthplace was unknown (unknown). Note that percentages have been rounded and may not total 100%.

We first examined the phylogenetic structure of our *M. tuberculosis* population and explored the association between lineage and our study variables. An MST revealed numerous large Euro-American clusters with distinct clades containing sizable clusters (Figure 1). Consistent with previous research [17], we found that lineage reflected birthplace (Supplementary Figure S2), and Canadian-born patients made up the majority in the Euro-American group (57.7%). Most MDR isolates (13 of 18) belonged to the East-Asian lineage (Supplementary Table S1). Disease site varied by lineage, and we found that the proportion of exclusively nonrespiratory tuberculosis was higher among patients with an Indo-Oceanic lineage (26.7%) compared with other lineages: East-Asian Indian (18.2%), East-Asian (12.6%), and Euro-American (10.4%) (*P <* .001). Patients with an Indo-Oceanic strain also had the highest proportion of respiratory disease with nonrespiratory involvement (Supplementary Table S2).

Clustering rates varied between lineages, with 54.5% of Euro-American, 43.3% of East-Asian, 33.8% of Indo-Oceanic, and 22.7% of East-African Indian isolates clustering. The 5 largest clusters belonged to the Euro-American lineage (Supplementary Table S3).

### Use of MIRU-VNTR Genotyping to Identify Discrete Subgroups Among Patients With Tuberculosis in BC

We next examined patient- and community-level risk factors driving clustering in BC. MIRU-VNTR revealed that, of 2290 isolates, 1319 (57.6%) were unique profiles, probably reflecting LTBI reactivation, whereas the remaining 42.4% were grouped into 189 clusters (2–70 patients per cluster), suggesting potential local transmission (Table 2). By means of the “n − 1” method [18] MIRU-VNTR estimated that 782 (34.1%) of infections could have resulted from local transmission. The median age of nonclustered individuals was higher (56 years) than that of clustered individuals (48 years; *P <* .001). Among male patients, 44.6% were clustered, versus 39.3% among female patients. Other factors for clustering included HIV status, drug use, and alcohol misuse (Table 3).

**Table 2.** Genotyping Results (24-Locus MIRU-VNTR), Including Genotype Clusters (n = 189) by Size and Frequency in British Columbia, 2005–2014^a^

Characteristic	No. (%)^b^
Isolates	
Unique genotype	1319 (57.6)
Clustered genotype	971 (42.4)
Clusters by size, No. of persons	
2	102 (54.0)
3	33 (17.5)
4	7 (3.7)
5–9	31 (16.4)
10–29	10 (5.3)
30–49	3 (1.6)
≥50	3 (1.6)

Abbreviation: MIRU-VNTR, mycobacterial interspersed repetitive-unit–variable-number tandem repeat.

^a^Clusters are defined as ≥2 patients with *Mycobacterium tuberculosis* infection who share an identical genotype.

^b^Percentages have been rounded and may not total 100%.

**Table 3.** Distribution and Univariable Analysis of Risk Factors Associated With *Mycobacterium tuberculosis* Genotypic Clustering Stratified by Birthplace in British Columbia, 2005–2014

Characteristic	Patients, No. (%)^a^		Clustered vs Unique, OR (95% CI)		
	Clustered	Unique	All Patients	Canadian Born	Foreign Born
Age, y					
0–14	16 (50.0)	16 (50.0)	1.3 (.6–2.6)	0.8 (.3–2.5)	0.7 (.2–2.3)
15–34	221 (44.2)	279 (55.8)	Reference	Reference	Reference
35–54	370 (52.6)	334 (47.4)	1.4 (1.1–1.8)	2.4 (1.4–4.2)	1.0 (.7–1.3)
55–74	237 (40.6)	347 (59.4)	0.9 (.7–1.1)	0.9 (.5–1.5)	0.9 (.6–1.1)
≥75	127 (27.0)	343 (73.0)	0.5 (.4–.6)	0.3 (.2–.6)	0.6 (.5–.9)
Sex					
Female	377 (39.3)	583 (60.7)	Reference	Reference	Reference
Male	593 (44.6)	736 (55.4)	1.2 (1.1–1.5)	1.1 (.7–1.6)	1.1 (.9–1.4)
Community type					
Metro	678 (38.7)	1075 (61.3)	Reference	Reference	Reference
Urban/rural	142 (42.8)	190 (57.2)	1.2 (.9–1.5)	0.7 (.4–1.1)	0.9 (.7–1.3)
Rural	126 (72.8)	47 (27.2)	4.3 (3.0–6.0)	2.1 (1.2–3.6)	0.8 (.4–1.8)
Remote	25 (78.1)	7 (21.9)	5.7 (2.4–13.2)	3.6 (.8–15.5)	1.7 (.4–7.7)
Birthplace					
Canada	453 (77.0)	135 (23.0)	7.8 (6.2–9.6)	…	…
Outside Canada	497 (30.2)	1148 (69.8)	Reference		
Disease site					
Respiratory	776 (43.9)	991 (56.1)	1.5 (1.2–1.9)	1.7 (.9–3.3)	1.0 (.8–1.3)
Nonrespiratory	125 (34.4)	238 (65.6)	Reference	Reference	Reference
Respiratory and nonrespiratory	70 (43.8)	90 (56.2)	1.5 (1.0–2.2)	2.1 (.8–5.9)	1.2 (.7–1.8)
Positive respiratory smear^b^	521 (45.2)	631 (54.8)	1.1 (.9–1.4)	1.6 (1.0–2.4)	0.9 (.7–1.1)
Cavitary disease	156 (49.5)	159 (50.5)	1.4 (1.1–1.8)	0.8 (.5–1.4)	1.3 (1.0–1.8)
HIV infected	66 (64.1)	37 (35.9)	2.6 (1.7–3.9)	1.6 (.8–3.1)	0.6 (.3–1.5)
Illicit drug use	112 (86.2)	18 (13.8)	10.3 (6.2–17.0)	2.7 (1.5–5.0)	3.8 (.9–16.1)
Alcohol misuse	97 (77.6)	28 (22.4)	5.6 (3.6–8.6)	2.7 (1.4–5.1)	1.4 (.6–3.2)
Material deprivation index					
Quintile 1 (least deprivation)	100 (36.6)	173 (63.4)	Reference	Reference	Reference
Quintile 2	148 (35.4)	270 (64.6)	0.9 (.7–1.3)	2.0 (.9–4.4)	0.9 (.6–1.4)
Quintile 3	196 (37.1)	333 (62.9)	1.0 (.8–1.4)	1.3 (.7–2.6)	1.0 (.7–1.5)
Quintile 4	220 (41.6)	309 (58.4)	1.2 (.9–1.7)	1.8 (.9–3.7)	1.2 (.8–1.8)
Quintile 5 (most deprivation)	224 (52.5)	203 (47.5)	1.9 (1.4–2.6)	2.3 (1.2–4.4)	1.1 (.7–1.7)

Abbreviations: CI, confidence interval, HIV, human immunodeficiency virus; OR, odds ratio.

^a^Percentages have been rounded and may not total 100%.

^b^“Other respiratory” sites (eg, pleura) were excluded.

Within the group of Canadian-born patients, the majority (77.0%) were in a cluster, compared with only 30.2% of foreign-born persons; indeed, the odds of belonging to a cluster were 7.8 higher for the Canadian born (95% CI, 6.2–9.6), Table 3. Interestingly, few clusters (16.4%) were exclusively Canadian born (Figure 1). When individuals were stratified by birthplace, risk factors for clustering followed similar trends between the Canadian born and the foreign born; however, the strength of association differed (Table 3). For example, both Canadian- and foreign-born persons residing in remote communities had increased odds of belonging to a cluster compared with individuals in metro areas, but odds were higher among the Canadian born (3.6. vs 1.7). Drug and alcohol use were also significantly associated with clustering in Canadian-born persons, and those living in areas of high material deprivation had 2.3 higher odds of belonging to a cluster (95% CI, 1.2–4.4).

### Use of MIRU-VNTR Genotyping to Identify Drivers of Large Transmission Clusters

Finally, we explored the differences between large clusters, typically representing outbreaks requiring public health intervention, and smaller clusters. Individuals in large clusters (≥10 persons) were more likely to be Canadian born (adjusted odds ratio, 3.3; 95% CI, 2.3–4.8), reside in a rural area (2.3; 1.2–4.5), or use drugs (2.0; 1.2–3.4) (Table 4).

**Table 4.** Multivariable Analysis of Factors Associated With Large and Small 24-Locus MIRU-VNTR Clusters in British Columbia, 2005–2014 (n = 971)^a^

Characteristic	Large vs Small, OR (95% CI)	Large vs Small, aOR (95% CI)^b^
Age, y		
0–14	0.9 (.3–2.6)	0.7 (.2–2.6)
15–34	Reference	Reference
35–54	1.4 (1.0–2.0)	1.2 (.8–1.8)
55–74	0.9 (.6–1.3)	1.1 (.7–1.8)
≥75	0.5 (.3–.8)	0.9 (.5–1.6)
Male sex	1.3 (1.0–1.7)	1.4 (1.0–1.9)
Community type		
Metro	Reference	Reference
Urban/rural	1.4 (.9–2.0)	0.9 (.6–1.5)
Rural	3.2 (2.1–4.9)	2.3 (1.2–4.5)
Remote	0.7 (.3–1.5)	0.5 (.2–1.4)
Canadian born	4.6 (3.5–6.1)	3.3 (2.3–4.8)
Illicit drug use	4.9 (3.1–7.8)	2.0 (1.2–3.4)

Abbreviations: aOR, adjusted odds ratio; CI, confidence interval; MIRU-VNTR, mycobacterial interspersed repetitive-unit–variable-number tandem repeat; OR, odds ratio.

^a^Large clusters were defined as ≥10 persons; small clusters, as <10 persons.

^b^Adjusted for age, sex, community type, birthplace, and drug use.

Among the 16 large clusters (Supplementary Table S3), 9 comprised predominantly Canadian-born individuals (≥87.1%), and the few foreign-born individuals within these clusters had a median time from immigration to active tuberculosis disease of 40 years (interquartile range, 25–49 years). In addition, for these foreign-born persons, where country of birth was known (n = 24), only 5 (20.8%) had emigrated from countries with a high tuberculosis burden [16]. Conversely, among the 7 large clusters comprising mainly foreign-born individuals, most individuals (89.9%) had emigrated from high-burden countries and had a significantly lower median time from immigration to active disease (12 years; interquartile range, 3–18 years) (*P <* .001).

## DISCUSSION

We describe the molecular epidemiology of tuberculosis in BC from 2005 through 2014 and demonstrate, using a near-complete (99.3%) isolate collection, that BC has notable strain diversity, with >1500 distinct MIRU-VNTR genotypes. The *M. tuberculosis* population structure reflects the global nature of BC’s residents. Migration to BC has been occurring for several centuries, first by predominantly European settlers and later with individuals from all over the world—especially from Asia [19]—which is reflected in the proportion of each lineage by region of birth. Clustering rates vary between lineages, with our largest clusters belonging to the Euro-American lineage, typical of what has been reported in European– and North American–born populations [17]. An MST revealed sizable clusters within the Euro-American lineage and distinct subgroups, probably reflecting a long history of migration to Canada and independent introduction of strains that have diversified and now circulate endemically, such as those introduced during the fur trade in previous centuries [20].

Different *M. tuberculosis* lineages have frequently been associated with phenotypic differences, such as propensity for drug resistance, varying pathogenicity, and tendencies toward specific disease sites [21, 22]. Indeed, we observed the bulk of MDR disease occurring in individuals with East-Asian strains, whereas individuals with Indo-Oceanic and East-African Indian lineages had higher odds of nonrespiratory disease and the lowest clustering rates, an observation in line with a large US study [21]. Given that nonrespiratory tuberculosis requires a high index of suspicion for diagnosis and commonly results in diagnostic delays and increased morbidity and mortality [23], our observation suggests that clinicians treating individuals who have emigrated from countries where Indo-Oceanic and East-African Indian strains circulate, might benefit from educational initiatives urging them to “think tuberculosis.”

Overall, we identified 189 clusters comprising 42.4% of the study isolates. Clustering rates previously reported from smaller studies in BC have varied substantially, with earlier work in the Greater Vancouver area reporting a much smaller clustering rate of 17.3% [6] and a study of Western Canadian provinces suggesting clustering from 0%-82% [10]. Given the near-complete sampling over a decade-long period that we undertook, our figure represents the most accurate estimate of genotype-level clustering for this setting.

Using the “n − 1” method [18], we estimated that 34.1% of our cases may be the result of local transmission, a figure identical to that of a study from London, United Kingdom [24], a city with a similarly large and ethnically diverse population. This is certainly an overestimation—reports directly comparing MIRU-VNTR to whole-genome sequencing (WGS) have shown that genotype-level identity does not always correspond to genomic distances that reflect recent, local transmission [25, 26], Indeed, we noted 2 large Indo-Oceanic clusters whose MIRU-VNTR match those of clusters reported elsewhere in Canada [25]. WGS yielded genomic distances incompatible with local transmission, suggesting that these clusters probably represent regionally endemic strains acquired in the country of origin rather than transmission within Canada [25]. Our future work includes sequencing of clustered isolates identified here to further refine our estimate of transmission, and will allow us to prioritize MIRU-VNTR clusters for investigation.

Where MIRU-VNTR is most likely to capture true local transmission is among the Canadian born. These individuals had nearly 8 times the odds of belonging to a cluster, and we identified multiple large clusters—2 already characterized by WGS [27–29], and most known to TB public health personnel and involving documented epidemiological links. In a New York City–area study, US-born residents were more likely to be involved in transmission clusters than foreign-born residents, with the authors concluding that transmission occurs almost exclusively within the American-born population [30]. However, in our study, nearly one-quarter of clusters involved both Canadian- and foreign-born individuals, suggesting that transmission probably occurs both across and within these populations. A 2014 systematic review of European tuberculosis found that the percentage of cases in “mixed” clusters ranged from 0% to 34.2% [31]; the extent to which this is occurring in BC will be revealed through genomic investigation.

Understanding where and among whom transmission is occurring permits targeted contact tracing and cluster investigation efforts, improved resource allocation, and interventions tailored to local epidemiology. In the current study, we found that though incidence was higher in metro areas, the odds of clustering were higher and cluster size was larger in rural and remote settings (Table 3 and Table 4), suggesting that local transmission dominates in low-density settings, whereas both local transmission and LTBI reactivation contribute to tuberculosis case counts in urban areas. Patient-level factors, including HIV infection, drug or alcohol use, or residence in a marginalized area, were all associated with increased odds of clustering, consistent with other studies [32–34]. All of this information could be used to develop a risk score for an individual contributing to onward transmission, based on both clinical and demographic factors and a strain’s specific genotype and lineage. Such a score could be used to prioritize patients for enhanced contact tracing and follow-up during therapy. In addition, our observation that nearly 70% of foreign-born persons have a unique genotype suggests that targeted LTBI screening is an important strategy for preventing the reactivation that is contributing to the bulk of tuberculosis diagnoses in BC.

Our data set included only a small number of MDR tuberculosis cases, the majority of which occurred in foreign-born individuals with East-Asian lineage isolates, a lineage known for its association with drug resistance [35]. With one exception—a known family transmission—MDR tuberculosis isolates did not show clustering by MIRU-VNTR, indicating that transmission most likely occurred before arrival in Canada. As immigrant numbers continue to rise in BC, with many persons arriving from regions with high rates of MDR tuberculosis, we are at risk of increased MDR tuberculosis, as reported in other low-incidence settings [36]. Thus, it is vital to have the molecular tools available to monitor the presence of drug-resistant strains and differentiate MDR tuberculosis caused by treatment failure from newly acquired MDR tuberculosis infection.

The present study does have some important limitations. As noted, although the discriminatory power of MIRU-VNTR is similar to that of restriction fragment length polymorphism (RFLP) analysis [37], it does not provide the necessary resolution to differentiate closely related isolates, particularly for non–Euro-American lineages [25, 26]. It has been suggested that Euro-American strains were overrepresented during method development, leading to a bias in the discriminatory power toward this lineage [38]. WGS can improve this resolution, which we plan to carry out in future work.

Second, an individual’s country of birth may not accurately reflect his or her movement. Although *M. tuberculosis* lineage often matches what we expect based on birthplace, some individuals may have lived in other countries before arrival in Canada, and may also travel after immigration. Some of our Canadian-born patients may have foreign-born parents, potentially increasing their risk of tuberculosis infection through household exposures and/or travel to their parents’ birthplace. This may account for some of the mixed Canadian-born/foreign-born clusters. Unfortunately, this level of detail is not included in most public health databases, precluding its analysis, but these scenarios are likely to be infrequent. What is clear is that “foreign born” is too broad a category, and a more refined definition would benefit tuberculosis surveillance efforts. Long-time residents of Canada with social risk factors comprise a very different group compared with recent immigrants, and should be viewed as a distinct group by tuberculosis programs.

Our study provides a benchmark against which we can measure future progress and offers new insight into the molecular epidemiology of tuberculosis in BC. This knowledge can be used to support new policy and practice as we move toward the ultimate goal of tuberculosis elimination, whether it be LTBI screening and prophylaxis in the foreign-born population or a risk score to stratify individuals’ risk of onward transmission. In a setting with declining tuberculosis incidence, contact network heterogeneity means that local pockets of transmission will exist [39], and identifying these quickly is critical to elimination efforts. Our finding around rural/remote transmission highlights these regions as hot spots for such pockets. We recommend better training of rural clinicians around recognizing tuberculosis, improved access to screening and treatment services, and the introduction of mobile technologies to facilitate a virtual clinic model [40]. Moreover, to limit the spread of infection, we recommend a lower threshold for extensive contact tracing in these regions. In conclusion, it is clear that a multipronged approach that includes targeted screening, treatment, and contact tracing informed by molecular epidemiology will have the greatest impact on tuberculosis rates in BC.

## Supplementary Data

Supplementary materials are available at *Clinical Infectious Diseases* online. Consisting of data provided by the authors to benefit the reader, the posted materials are not copyedited and are the sole responsibility of the authors, so questions or comments should be addressed to the corresponding author.

## Supplementary Material

Supplementary Data

## Notes

***Acknowledgments*.** We acknowledge the tuberculosis/mycobacteriology staff of the BCPHL, British Columbia Centre for Disease Control (BCCDC), for providing the tuberculosis isolates for this study, and Fay Hutton of BCCDC’s tuberculosis services for her assistance with Integrated Provincial Health Information System data extraction. Thank you to Sunny Mak (BCCDC senior medical geographer) for his assistance with the Canadian marginalization index data.

***Financial support*.** This work was supported by the BCCDC Foundation for Population and Public Health, the British Columbia Lung Association, the University of British Columbia (4-year doctoral fellowship to J. L. Guthrie), the Canadian Institutes of Health Research (doctoral research award to J. L. Guthrie), Killam Trusts (doctoral scholarship to J. L. Guthrie), the Michael Smith Foundation (scholar awards to J. J. and J. L. Gardy), and the Canada Research Chairs Program (J. L. Gardy).

***Potential conflicts of interest.*** All authors: No reported conflicts. All authors have submitted the ICMJE Form for Disclosure of Potential Conflicts of Interest. Conflicts that the editors consider relevant to the content of the manuscript have been disclosed.
