# Supplementary material for: Molecular Epidemiology of Tuberculosis in British Columbia, Canada: A 10-Year Retrospective Study
Source: Clin Infect Dis. 2017 Oct 21;66(6):849–56. doi: 10.1093/cid/cix906 (PMC5850024; doi:10.1093/cid/cix906)
Supplement: Supplementary Data [file cix906_suppl_supplementary_data.pdf]

SUPPLEMENTARY DATA

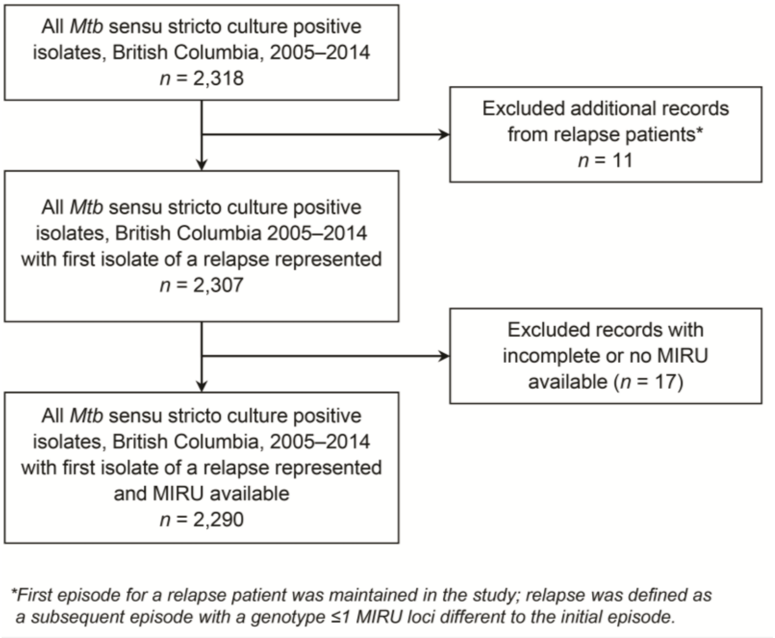

Figure S1. Study inclusion/exclusion criteria.

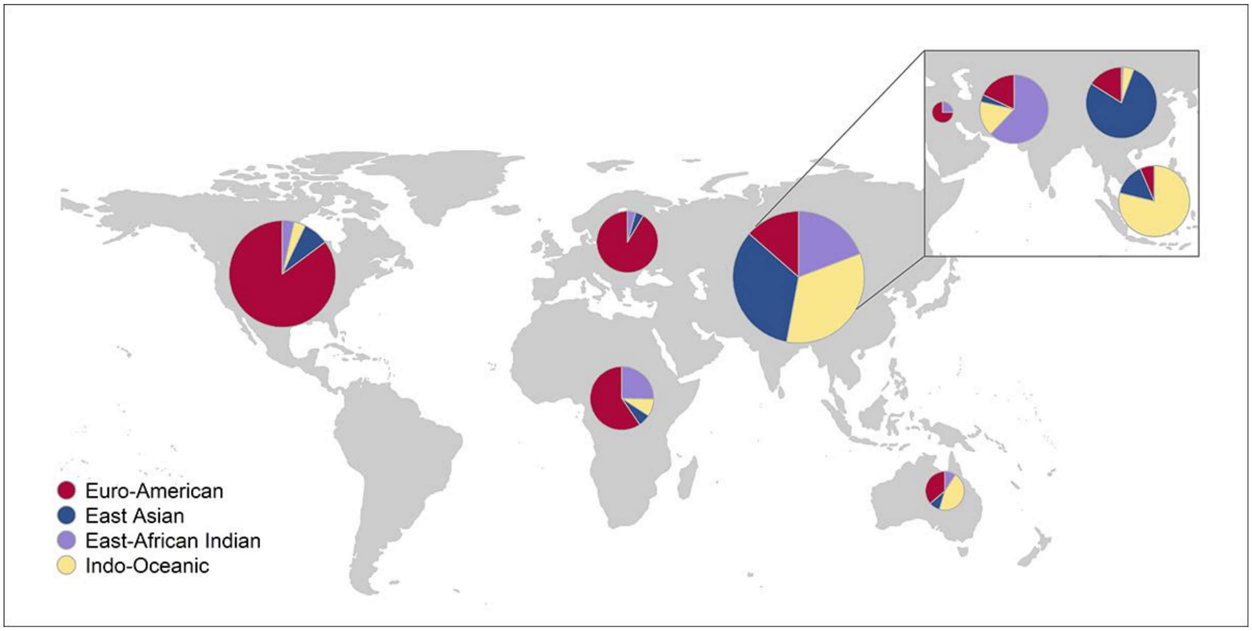

Figure S2. Relative frequency of *Mycobacterium tuberculosis* sensu stricto lineages by patients' continent of birth. The inset map separates the *Mtb* lineages of Asian-born patients into the relative frequency observed within each Asian sub-continent. Pie chart areas are scaled relative to the number of cases.

Table S1. Characteristics of multi-drug resistant *Mycobacterium tuberculosis* patients in British Columbia, 2005–2014 (*n*=18).

Birth Sub-Continent	Region*	Case Type	Disease Site†	Lineage	24MIRU Cluster‡
North America	GVR	New	Resp.	East-Asian	Yes
North America	GVR	New	Resp.	East-Asian	Yes§
East Asia	GVR	New	Resp.	East-Asian	Yes§
East Asia	GVR	New	Resp.	East-Asian	
East Asia	GVR	Retreatment	Resp.	East-Asian	Yes
East Asia	GVR	Retreatment	Resp.	East-Asian	
South-Central Asia	GVR	New	Resp.	East-African-Indian	Yes
South-Eastern Asia	GVR	Retreatment	Resp.	Indo-Oceanic	
South-Eastern Asia	GVR	New	Non-Resp.	Indo-Oceanic	
South-Eastern Asia	GVR	New	Non-Resp.	Indo-Oceanic	Yes
South-Eastern Asia	GVR	New	Resp. + Non-Resp.	Indo-Oceanic	
Northern Europe	GVR	New	Resp.	East-Asian	Yes
East Asia	GVR	Retreatment	Resp.	East-Asian	
East Asia	GVR	New	Resp.	East-Asian	Yes
East Asia	non-GVR	Retreatment	Resp.	East-Asian	
East Asia	GVR	Retreatment	Resp.	East-Asian	
South-Eastern Asia	GVR	New	Resp.	East-Asian	
South-Eastern Asia	GVR	New	Resp.	East-Asian	

**GVR: Greater Vancouver Region.*
†*Resp. = respiratory; Non-Resp. = non-respiratory.*
‡*Yes indicates that the isolate belongs to a 24MIRU cluster.*
§*Same cluster; known transmission event.*

Table S2. *Mycobacterium tuberculosis* sensu stricto lineages by anatomical disease site in British Columbia, 2005–2014.*

	No. Patients (%)			NRTB vs. RTB
Lineage	RTB	NRTB	RTB+NRTB	**OR (95%CI)**
Euro-American	723 (83.0)	91 (10.4)	57 (6.5)	Reference
East-Asian	456 (81.9)	70 (12.6)	31 (5.6)	1.2 (0.9–1.7)
East-African Indian	252 (76.4)	60 (18.2)	18 (5.5)	1.9 (1.3–2.7)
Indo-Oceanic	336 (63.2)	142 (26.7)	54 (10.2)	3.4 (2.5–4.5)

RTB—exclusively respiratory; NRTB—exclusively non-respiratory; RTB+NRTB—respiratory+non-respiratory; OR—odds ratio; CI —confidence interval.
**Percentages have been rounded and may not total to 100%.*

Table S3. Characteristics of 24-locus MIRU–VNTR clusters comprised of ≥ 10 individuals, displayed as clusters that were predominantly Canadian- or foreign-born: British Columbia, 2005–2014.

Cluster ID	Cluster Size	Predominant Birthplace* (%)	Median Age (IQR) years	Gender M:F	Predominant Community Type (%)	Lineage
Canadian–born						
MClust-002	70	Canada (88.1)	50 (43–57)	13.0	Metro (78.6)	Euro-American
MClust-012	64	Canada (87.1)	48 (40–56)	3.0	Metro (79.7)	Euro-American
MClust-001	56	Canada (96.4)	40 (29–48)	0.8	Rural (75.0)	Euro-American
MClust-003	39	Canada (97.4)	45 (29–50)	1.8	Rural (56.4)	Euro-American
MClust-008	36	Canada (91.7)	41 (35–55)	1.1	Metro (83.3)	Euro-American
MClust-035	17	Canada (88.2)	39 (32–58)	1.7†	Metro (82.4)	East-Asian
MClust-052	17	Canada (94.1)	49 (46–53)	3.2	Metro (82.4)	Euro-American
MClust-134	13	Canada (100.0)	59 (46–68)	3.3	Rural (84.6)	Euro-American
MClust-055	10	Canada (100.0)	38 (31–43)	1.5	Urban/Rural (70.0)	Euro-American
Foreign-born						
MClust-011	34	Philippines (97.0)	41 (31–50)	2.1	Metro (76.5)	Indo-Oceanic
MClust-021	25	Philippines (100.0)	50 (31–54)	0.8	Metro (84.0)	Indo-Oceanic
MClust-038	16	China/Hong Kong (87.5)	76 (64–81)	1.3	Metro (100.0)	East-Asian
MClust-187	16	China/Hong Kong (80.0)	66 (47–87)	0.6	Metro (100.0)	East-Asian
MClust-149	13	India (84.6)	59 (34–79)	0.9	Metro (61.5)	East-African Indian
MClust-046	12	India (75.0)	71 (64–78)	2.0	Metro (91.7)	East-African Indian
MClust-032	11	*Mixed*‡	54 (48–82)	1.2	Metro (100.0)	East-Asian

IQR—interquartile range.

**Information for birthplace was unknown for 9 patients; percentage represents those with complete data.*

†Not included in the ratio, 1 transgender/gender unknown individual.

‡Predominantly East Asian and South-East Asian countries
